# Personality change after Internet-delivered cognitive behavior therapy for depression

**DOI:** 10.7717/peerj.39

**Published:** 2013-02-19

**Authors:** Robert Johansson, Caroline Lyssarides, Gerhard Andersson, Andréas Rousseau

**Affiliations:** 1 Department of Behavioural Sciences and Learning, Linköping University, Linköping, Sweden; 2 Psychiatric Clinic, University Hospital of Linköping, Linköping, Sweden; 3 Swedish Institute for Disability Research, Linköping University, Linköping, Sweden; 4 Department of Clinical Neuroscience, Psychiatry Section, Karolinska Institutet, Stockholm, Sweden

**Keywords:** Depression, TCI, Personality, Temperament, Character, Internet, Cognitive behavior therapy

## Abstract

**Background.** The Temperament and Character Inventory (TCI) by Cloninger is a widely used instrument to measure personality dimensions. Two dimensions of the TCI, Harm avoidance (HA) and Self-Directedness (SD), are known to be influenced by depressed mood. This study investigated changes in HA and SD after 10 weeks of Internet-delivered cognitive behavior therapy (ICBT) in a sample of clinically depressed subjects (*N* = 108). Differences in personality changes among treatment responders and non-responders were also investigated. Exploratory investigations on changes for other TCI dimensions, were also conducted.

**Methods.** Depressed subjects were randomized either to ICBT or to a moderated online discussion group, which served as an active control group. The interventions lasted for 10 weeks. TCI was measured at baseline and after treatment. Depressive symptoms were assessed using the Beck Depression Inventory-II.

**Results.** There were significant changes on HA and SD after ICBT. However, when comparing post-treatment HA and SD to the control, no differences were found. Among responders, larger changes compared to non-responders were found in HA and in SD, as well as in Cooperativeness.

**Conclusions.** The study showed that HA and SD changed after ICBT. The changes in personality seem related to improvement in depression rather than a direct effect of ICBT.

## Introduction

Major depression disorder (MDD) affects millions of people worldwide each year and often recurs if left untreated. Despite effective treatments being available, only about 50% of cases receive adequate treatment (Ebmeier, Donaghey & Steele, 2006). A person’s personality has been proposed to be a vulnerability factor for developing depression (i.e., a trait aspect), but also to be affected by current mood (i.e., a state aspect), in particular sadness (Farmer et al., 2003; Halvorsen et al., 2009). Clinically, it is therefore a challenge to assess personality function during a current depressive episode.

Cloninger’s Temperament and Character Inventory (TCI) is a well-used self-report measure of personality that captures both state and trait aspects when used during depressive episodes (Cloninger, Svrakic & Przybeck, 1993). In Cloninger’s model, temperament is separated into four different higher-order scales: Novelty Seeking (NS), Harm Avoidance (HA), Reward Dependence (RD) and Persistence (P), while character is divided into three scales: Self-Directedness (SD), Cooperativeness (C) and Self-Transcendence (ST).

Some dimensions of the TCI seem to be more influenced by depressive states. Hansenne et al. (1999) found that among patients with depression, the HA, SD and C dimensions of TCI were related to the severity of depression. Similarly, Hirano et al. (2002) found that HA, SD and C were associated with depression severity and that these dimensions changed during antidepressant treatment among treatment responders. More recently, Spittlehouse et al. (2010) also found that changes in SD and HA after antidepressant treatment and psychotherapy were related to improvements in depression. A recent meta-analysis on depression and the temperament dimensions of the TCI, provided further evidence that HA can change during treatment, with a mean pre to post decrease of 2.12 (95% CI: 1.39–2.86) and 3.13 (95% CI: 1.88–4.38) among responders (Kampman & Poutanen, 2011). In summary, research shows that the higher-order scales HA and SD consistently seem to be affected by both pharmacological and psychotherapeutic treatments, supporting the state aspect of these dimensions of personality. Results on other dimensions on the TCI have been more inconsistent.

Certain personality traits have been proposed to act as vulnerability factors for the development of depression (Cloninger, Svrakic & Przybeck, 2006; Farmer et al., 2003). Individuals with high HA are emotionally anxiety-prone which has been suggested to be an independent risk factor (Cloninger, Svrakic & Przybeck, 2006). In contrast, high SD has been shown to be protective against depression (Cloninger, Svrakic & Przybeck, 2006). SD concerns being responsible, purposeful and resourceful. A combination of both high HA and low SD has been shown to be a particular risk factor for the development of depression (Cloninger, Svrakic & Przybeck, 2006; Farmer et al., 2003). One study that lends support for trait aspects of personality is the study by Tang et al. (2009). The authors used the NEO-Five Factor Inventory (Costa & Mccrae, 1992) for the assessment of personality in subjects with MDD who were randomized to selective serotonin reuptake inhibitors (SSRIs), cognitive therapy (CT) or placebo. Tang et al. (2009) showed that SSRIs, compared to placebo, had an effect on Neuroticism (which according to Bagby et al. (2008) can be considered equivalent to HA) and Extraversion (similar to NS and RD; (Bagby et al., 2008)), even after controlling for improvements in depression. CT also had, compared to placebo, an effect on Neuroticism and Extraversion. However, when accounting for depression improvement the effect of CT on Neuroticism did not remain significant. In summary, Tang et al. (2009) provided evidence that personality change after SSRI treatment seem not to be merely a consequence of change in depressive states. On the contrary, an earlier study by Agosti & McGrath (2002) did not find any treatment-specific effects on personality, as measured by the TCI. The study investigated the effects of fluoxetine, imipramine and placebo in a sample of outpatients with atypical depression. However, the authors did find that responders decreased more in HA than non-responders. To our knowledge, no other placebo-controlled randomized trials exist in which treatment effects on TCI dimensions are investigated in depression.

During the last decade, guided Internet-delivered cognitive behavior therapy (ICBT) has proved to be a useful way of providing psychotherapy for a variety of mental disorders (Andersson, 2009), including panic disorder (Carlbring et al., 2001), social phobia (Andersson et al., 2006), generalized anxiety disorder (Paxling et al., 2011) and MDD (Andersson et al., 2005; Johansson & Andersson, 2012; Meyer et al., 2009; Perini, Titov & Andrews, 2009). In MDD, it has been shown that ICBT with support (e.g., e-mail support) is about as effective as traditional CBT (Andrews et al., 2010; Cuijpers et al., 2010). Obvious major advantages are easy access and cost-effectiveness aspects. While the evidence for ICBT for MDD is strong, we do not know of any studies investigating the effect of ICBT for MDD on personality. There is, however, one study investigating how the NEO-Five Factor Inventory could predict outcome in ICBT for sub-threshold depression, where the authors found that low neuroticism could predict better outcome in ICBT, but also in a CBT-based group intervention (Spek et al., 2008).

In the present study, personality was assessed using TCI in subjects with MDD before and after 10 weeks of ICBT as part of a study investigating the effects of ICBT compared to a control condition (a moderated online discussion group). Details of the clinical trial can be found elsewhere (Johansson et al., 2012). The aims of the present study were twofold. Firstly, to investigate the effects of ICBT on personality in subjects with MDD and to compare these effects to those resulting from an active control. Secondly, to compare differences in changes in personality dimensions between treatment responders and non-responders. Based on the literature, a decrease in HA and an increase in SD were expected within the ICBT group. A difference in post-treatment HA and SD was also expected when comparing the treatment to control, as well as when comparing responders to non-responders. Exploratory investigations on other TCI dimensions were also conducted.

## Materials & methods

### Participants and recruitment

This study used a subsample of participants from the study by Johansson et al. (2012). Participants were recruited from the community. The original study was approved by the Regional Ethics Board of Linköping, Sweden (case number M98-09). Written informed consent was obtained from all participants by surface mail.

The selection process started with an online screening, with demographic questions and online versions of measures of depression, anxiety and quality of life (described below). In addition, the screening contained the revised Swedish version of the Temperament and Character Inventory (TCI; (Cloninger, Svrakic & Przybeck, 1993)). The results from the online screening were later used as pre-treatment assessment for those included in the study.

Subjects who had completed the online screening were contacted by telephone for an interview using the Structured Clinical Interview for DSM-IV – Axis I disorders, clinical version (SCID-I-CV; (First et al., 1997)). To be included in the study, the participant had to fulfill the following criteria: (a) being at least 18 years; (b) meeting DSM-IV criteria for major depression, with a current acute episode of depression or an episode in partial remission; c) having access to a computer and Internet; (d) not participating in other psychological treatment for depression at the time; (e) if on psychopharmacological treatment, unchanged dosage for the last 3 months; (f) a total of 15–35 on the Montgomery Åsberg Depression Rating Scale-Self Rated (MADRS-S; (Svanborg & Åsberg, 1994)); (g) less than 5 points on question nr 9 on MADRS-S (suicidal ideation); (h) not having severe comorbidity that otherwise better could be treated in regular psychiatric care (e.g. severe bipolar disorder, schizophrenia or alcohol or drug abuse).

Out of the 121 who met inclusion criteria (a–h), 114 completed the TCI at post-treatment. Six subjects were finally excluded because of non-valid TCI data (answered “yes” on question nr 230: “I have not been totally honest in this questionnaire”). Thus TCI personality data were available for 108 subjects. A complete demographic description of the participants is given in Table 1.

**Table 1:** Demographic description of the participants.

		ICBT treatment	Control group	Total
Gender	Female	51 (72.9%)	27 (71.1%)	78 (72.2%)
	Male	19 (27.1%)	11 (28.9%)	30 (27.8%)
Age	Mean (SD)	44.0 (12.7)	45.1 (11.9)	44.4 (12.3)
	Min–Max	20–70	21–75	20–75
Marital status	Married or co-habiting	33 (47.2%)	22 (57.9%)	55 (50.9%)
	Other	37 (52.9%)	16 (42.1%)	53 (49.1%)
Educational level	College or university, completed	34 (48.6%)	21 (55.3 %)	55 (50.9%)
	College or university, ongoing	14 (20.0%)	2 (5.3%)	16 (14.8%)
	Other	22 (31.4%)	15 (39.5%)	37 (34.3%)
Employment status	Employed	43 (61.4%)	29 (76.3%)	72 (66.7%)
	Other	27 (38.6%)	9 (23.7%)	36 (33.3%)
Medication	No experience	23 (32.9%)	11 (28.9%)	34 (31.5%)
	Prior experience	30 (42.9%)	12 (31.6%)	42 (38.9%)
	Present	17 (24.3%)	15 (39.5%)	32 (29.6%)
Psychological treatment	No experience	26 (37.1%)	16 (42.1%)	42 (38.9%)
	Prior experience	40 (57.1%)	19 (50.0%)	59 (54.6%)
	Present	4 (5.7%%)	3 (7.9%)	7 (6.5%)
Depression	In acute episode	50 (71.4%)	28 (73.7%)	78 (72.2%)
	In partial remission	20 (28.6%)	10 (26.3%)	30 (27.8%)
Comorbidity	Social fear	21 (30.0%)	8 (21.1%)	29 (26.9%)
	Worry	16 (22.9%)	10 (26.3%)	26 (24.1%)
	Panic	12 (17.1%)	4 (10.5%)	16 (14.8%)
	Any anxiety	38 (54.3%)	19 (50.0%)	57 (52.8%)

### Assessments of depression and personality

All participants were assessed before therapy and at the time of termination. The measures were administered via the Internet, which has been shown to be a valid format for questionnaires regarding depression and anxiety (Carlbring et al., 2007; Holländare, Andersson & Engström, 2010). The principal measure of depression in this study was the Beck Depression Inventory-II (BDI-II; (Beck, Steer & Brown, 1996)). In the original study, secondary measures of depression (MADRS; (Svanborg & Åsberg, 1994)), anxiety measured by the Beck Anxiety Inventory (BAI; (Beck et al., 1988)) and quality of life measured by the Quality of Life Inventory (QOLI; (Frisch et al., 1992)) were also administrated. Details can be found in Johansson et al. (2012).

Personality was assessed using the Swedish version of the Temperament and Character Inventory (TCI), a 238-item, self-administered questionnaire, with measurements of seven separate personality scales. All questions were given as true/false statements. The TCI is scored on four temperament scales labeled Novelty Seeking (i.e., impulsive versus rigid), Harm Avoidance (i.e., anxious versus risk-taking), Reward Dependence (i.e., approval seeking versus aloof) and Persistence (i.e., overachieving versus underachieving). Temperament refers to individual differences in the sensitivity to specific environmental stimuli and the behavioral responses to those stimuli (Keltikangas-Järvinen, Ravaja & Viikari, 1999). Three character scales exists labeled Self-Directedness (executive functions, such as being problem-solving, self-confident and purposeful), Cooperativeness (relating to others, such as being tolerant, forgiving and helpful) and Self-Transcendence (judicial functions, such as being intuitive, judicious and aware) (Cloninger, Svrakic & Przybeck, 2006). Detailed descriptions of these scales are available elsewhere (Cloninger, Svrakic & Przybeck, 1993; Cloninger, 1994).

### Interventions

In the original study, subjects were randomized to one of three interventions: Individually tailored ICBT treatment, standardized (non-tailored) ICBT or participation in a moderated discussion group with a focus on depression. All interventions lasted for 10 weeks. In this specific study, the ICBT groups are considered as one, as no differences in personality change were expected between the ICBT interventions. Summaries of the interventions are given below. Details can be found in Johansson et al. (2012).

### Internet-delivered cognitive behavior therapy

There were 70 participants who were randomized to one of the two variants of ICBT. Both interventions were given as guided self-help, with weekly treatment modules and a therapist contact similar to e-mail carried out in a secure online environment. The ICBT interventions contained components from typical face-to-face CBT, including introduction to CBT, psychoeducation on depression, cognitive restructuring and relapse prevention. In addition, the standardized ICBT group received modules on behavioral activation and material on sleep management and general health advice. The tailored ICBT group received an individualized set of modules that targeted comorbidity with depression, such as anxiety, worry and stress.

### Moderated online discussion group

The active control group (which contained 38 participants) participated in a moderated discussion group on the Internet for 10 weeks. The members were encouraged by the moderator to engage in the discussion of different topics that were presented each week, all related to MDD and the treatment of depression. The choice to use a discussion group instead of a waiting list was made for ethical reasons. While it has been found that online support groups can have a small effect (Griffiths, Calear & Banfield, 2009), we expected that this effect would be small or non-existing. A few weeks after the ICBT groups had finished their treatment, the control group received the standardized treatment. This was mainly done for ethical reasons and data from that treatment is not reported in this study.

### Sample characteristics and classification of responders

The sample of 108 participants contained 73% women, averaging 44.4 years in age (range 20–75; sd = 12.3). At baseline, there were no significant differences between the ICBT and control in TCI scores and depression severity (all *t*’s < 1.36 and all *p*’s > .18).

Subgroups were formed based on treatment response. A subject was defined as a responder if his/her BDI-II-score decreased with at least 50% from pre-treatment to post-treatment. This definition is similar with the definition of responders used in previous clinical trials on depression (e.g. DeRubeis et al., 2005; Dimidjian et al., 2006). Importantly, participants from both the ICBT group and the active control group could be classified as a responders. There were no significant baseline differences between responders and non-responders, neither regarding level of depression (*t*(106) = 1.32, *p* = .19) nor any TCI dimension (all *t*’s < 1.83, all *p*’s > .07).

### Data analyses

Significance testing of within-group effects was conducted using repeated measures ANOVA. Mean differences between ICBT/control and responders/non-responders were analyzed using an analysis of variance controlling for pre-treatment differences, age and gender (ANCOVA). In all ANCOVA analyses, the effect of ICBT, treatment response, their interaction, age and gender were included. All analyses were made using SPSS 19 (SPSS, Inc., Chicago, IL).

Mean differences between ICBT/Control (a) and Responders/Non-responders (b) at post-treatment were analyzed using an analysis of variance controlling for pre-treatment differences (ANCOVA). In all ANCOVA analyses, the effect of ICBT, treatment response, their interaction, age and gender were included.

## Results

### Changes in symptoms of depression

Table 2 shows pre- and post-treatment scores on the BDI-II for the ICBT group and the control group. The ICBT group had a statistically significant lower level of depression than the control group at post-treatment (*F*(1, 101) = 5.3, *p* < .05). There was also a difference between responders and non-responders at post-treatment (*F*(1, 101) = 96.8, *p* < .001). Further details on the effect of the ICBT treatment can be found in Johansson et al. (2012).

**Table 2:** Mean pre-treatment and post-treatment TCI scores by treatment group and response status, and test-statistics for within- and between-group effects.

	ICBT (*n* = 70)			Control (*n* = 38)			Responders (*n* = 35)			Non-responders (*n* = 73)					
	Pre	Post	Within-group	Pre	Post	Within-group	Pre	Post	Within-group	Pre	Post	Within-group	ICBT^(a)^	Response^(b)^	ICBT × Response
	Mean (sd)		*F*(1, 69)	Mean (sd)		*F*(1, 37)	Mean (sd)		*F*(1, 34)	Mean (sd)		*F*(1, 72)	*F*(1, 101)	*F*(1, 101)	*F*(1, 101)
BDI-II	25.7 (7.9)	15.3 (9.8)	72.6^***^	26.3 (8.2)	21.6 (9.6)	12.9^***^	24.4 (7.6)	6.31 (3.9)	265.0^***^	26.6 (8.2)	22.9 (7.5)	18.1^***^	5.28^*^	96.8^***^	1.44
NS	20.2 (6.4)	21.6 (6.4)	12.3^***^	19.6 (5.9)	20.2 (5.9)	1.31	19.9 (5.8)	21.1 (5.8)	4.51^*^	20.0 (6.4)	21.1 (6.5)	7.93^**^	0.41	0.03	0.94
HA	24.1 (6.0)	21.1 (6.5)	39.1^***^	24.2 (5.8)	22.5 (5.9)	4.70^*^	23.1 (6.6)	18.4 (6.3)	42.7^***^	24.6 (5.5)	23.1 (5.8)	10.3^**^	0.00	16.0^***^	0.89
RD	15.0 (3.7)	15.5 (3.9)	2.66	15.6 (3.4)	15.1 (3.4)	2.15	15.7 (4.2)	16.2 (3.8)	1.05	15.0 (3.3)	15.0 (3.7)	0.02	4.62^*^	0.06	1.85
P	4.23 (2.3)	4.39 (2.1)	0.94	4.82 (2.0)	4.21 (2.0)	6.86^*^	4.51 (2.39)	4.74 (2.1)	0.82	4.40 (2.1)	4.12 (2.1)	2.93	5.06^*^	0.12	1.62
SD	20.0 (7.4)	24.1 (8.9)	27.1^***^	21.2 (7.1)	23.1 (7.8)	3.69	21.6 (7.3)	29.1 (5.9)	36.9^***^	19.9 (7.3)	21.2 (8.4)	5.14^*^	0.00	23.4^***^	0.02
C	30.8 (5.7)	32.0 (5.8)	5.23^*^	30.7 (6.1)	30.8 (5.9)	0.07	32.2 (4.8)	33.9 (4.4)	8.46^**^	30.1 (6.2)	30.4 (6.2)	0.59	0.12	4.38^*^	0.26
ST	10.2 (5.5)	10.7 (5.3)	1.05	11.8 (5.6)	11.1 (6.2)	0.97	11.9 (5.4)	12.3 (5.3)	0.33	10.3 (5.6)	10.1 (5.7)	0.06	0.23	0.58	0.15

**Notes.**

* *p* < 0.05.

** *p* < 0.01.

*** *p* < 0.001.

Within-group analyses were conducted using repeated measures ANOVA. Mean differences between ICBT/Control ^(a)^ and Responders/Non-responders ^(b)^ at post-treatment were analyzed using an analysis of variance controlling for pre-treatment differences (ANCOVA). In all ANCOVA analyses, the effect of ICBT, treatment response, their interaction, age and gender were included.

*Abbreviations:* ICBT: Internet-based cognitive behavior therapy; BDI-II: Beck Depression Inventory-II; NS: Novelty Seeking; HA: Harm Avoidance; RD: Reward Dependence; P: Persistence; SD: Self-Directedness; C: Cooperativeness; ST: Self-Transcendence; Pre: Pre-treatment; Post: Post-treatment; sd: standard deviation.

### Personality changes within and between ICBT and the control group

TCI-scores at baseline and after 10 weeks for the ICBT group and the control group are presented in Table 2. Baseline scores on the BDI-II or TCI did not differ between groups. In both the ICBT group and the control group, there was a significant decrease in HA (*F*(1, 69) = 39.1, *p* < .001 and *F*(1, 37) = 4.70, *p* < .05, respectively). For SD, there was a significant increase in the ICBT group (*F*(1, 69) = 27.1, *p* < .001), but not in the control condition.

In the treatment group, there were also significant increases in NS and C. Despite non-significant within-group changes in these dimensions in the control group, no differences between ICBT and control were observed at post-treatment.

For RD there was a non-significant increase in the ICBT group and non-significant decrease in the control group, resulting in a significant difference between ICBT and control at post-treatment (*F*(1, 101) = 4.62, *p* < .05). For P, there was an unexpected significant decrease in the control group, but not in the ICBT group. The post-treatment difference between ICBT and control on this dimension was also significant (*F*(1, 101) = 5.06, *p* < .05).

### Personality changes in responders compared to non-responders

Of the 108 participants, 35 (32.4%; 41.4% from ICBT and 15.8% from control) were classified as responders. TCI scores and symptom scores at baseline and after 10 weeks for responders and non-responders can be seen in Table 2. Baseline scores on the BDI-II and TCI did not differ between responders and non-responders.

As seen in Table 2, there were significant post-treatment differences between responders and non-responders on several TCI dimensions. This included HA (*F*(1, 101) = 16.0, *p* < .001), SD (*F*(1, 101) = 23.4, *p* < .001) and C (*F*(1, 101) = 4.38, *p* < .05).

## Discussion

To the best of our knowledge, this is one of the first randomized controlled trials which, in a sample with MDD, evaluates treatment effects on personality measured by the TCI. This trial is probably also the first that investigates personality changes after Internet-based cognitive behavior therapy for depression. The primary aim of the study was to study the effects of ICBT on HA and SD. The secondary aim was to investigate differences between treatment responders and non-responders on HA and SD. Effects on other TCI dimensions were explored, both among responders and in the entire sample.

As expected, personality changes were observed in HA and SD within the ICBT group. However, no differences in these TCI dimensions were observed at post-treatment when comparing ICBT and the control group. Among responders, there were larger changes on HA and SD than among non-responders. Exploratory analyses also confirmed this on the C dimension.

As seen in Table 2, the effect of ICBT on HA was 3.0 raw scores. This is above the overall mean difference presented in the meta-analysis by Kampman & Poutanen (2011), where the overall change of HA after treatment was 2.12 (95% CI: 1.39–2.86). The comparison of the effect of ICBT on SD to results from other treatment studies is more exploratory, as no formal meta-analysis of this seems to exist. However, the change of 4.1 SD scores after ICBT, seems similar to the change in SD after psychotherapy observed by Spittlehouse et al. (2010). In all, our study provides no indications that ICBT is less effective than other treatments for depression, regarding personality change in the HA and SD dimensions.

Larger effects than expected were found within the control group, with a substantial amount of responders (15.8%) and a decrease of 1.7 raw scores on HA. This and the absence of post-treatment differences between ICBT and control on HA and SD, indicates that the changes in these TCI dimensions are to be attributed to improvement in mood, i.e. the changes are not a cause of treatment in itself, as presented by Spittlehouse et al. (2010). These results are in line with Agosti & McGrath (2002) where no change in personality was found after treatment with antidepressants when comparing to placebo.

Among the responders, there were effects on HA and SD when compared to non-responders. This difference in means was 4.7 HA scores which is higher than the overall mean difference of 3.13 (95% CI: 1.88–4.38) among responders, as presented by Kampman & Poutanen (2011). Exploratory analyses also found an effect of treatment response on the C dimension. In conclusion, the results regarding responders are the exact same findings as in Hirano et al. (2002) where changes among responders were found in these three TCI dimensions. The findings also, in part, parallel Agosti & McGrath (2002) who found an effect on HA among responders.

Exploratory analyses also found a difference in post-treatment difference in RD and P between ICBT and control. In contrast to other findings in this study, these changes seem to be a consequence of specific ICBT effects, i.e. the differences remains when controlling for depression. This is somewhat in line with the meta-analysis by Kampman & Poutanen (2011), where a small significant overall increase of 0.59 (95% CI: 0.16–1.02) in RD scores was found after treatment. However, no significant overall change in P was observed by Kampman & Poutanen (2011). Furthermore, the result regarding RD are in part similar to the finding by Tang et al. (2009) on that cognitive therapy had a true effect on Extraversion (which has similarities to RD, as well as to NS; (Bagby et al., 2008)). A further interpretation of our findings in these dimensions is outside the scope of this study. However, future research is warranted to explain these findings.

Several limitations of the present study are acknowledged. Firstly, the study length was set to 10 weeks which is a short period of time when studying personality change, especially trait changes. Thus, ICBT-specific effects on personality could have been missed since change may take longer than 10 weeks to achieve. However, the Tang et al. (2009) study found CBT-specific effects on Extraversion and effects of SSRI on Neuroticism and Extraversion after only 8 weeks of treatment, indicating an adequate length of the ICBT treatment. Secondly, the control group was active, mainly because of ethical concern, and had substantial within-group effects. This may have resulted in that no specific ICBT effects could be identified. While the current scenario provided an opportunity to control for depression, it might also have covered true differences between ICBT and a group receiving no treatment. Thirdly, this research may not be generalizable to all individuals with major depression, since this study only included participants with mild to moderate depression. Fourthly, a methodological limitation is acknowledged in that no observer-rated depression symptom assessments were conducted. Finally, we used an online version of the TCI. While the Internet has shown to be a valid format for questionnaires regarding depression and anxiety (Carlbring et al., 2007; Holländare, Andersson & Engström, 2010), we do not know of any study that administrates the TCI online in a clinical trial. Even if no indications exist that this could bias the results, it is acknowledged as a limitation. Further research regarding validation of the TCI in the Internet format is warranted.

There are some implications of this study. The ICBT intervention seems as effective as other treatments in changing the HA and SD dimensions, but that the effects do not remain when comparing the treatment group to the control. One implication of this is that the present study adds to the empirical base on personality changes in SD and HA (e.g. Kampman & Poutanen, 2011; Spittlehouse et al., 2010). Our study supports the state effect hypothesis, and the results from our study open up for a discussion on whether existing non-controlled studies on personality change should be interpreted as merely state effects or not.

The results from our study could be interpreted such that ICBT may not contain any working mechanisms beyond those available in the discussion group (which does not contain any specific interventions). While the overall high efficacy of ICBT for MDD seems to contradict this reasoning, one implication of the present study is that it makes explicit that more research on the exact workings of ICBT is clearly needed.

Finally, this study may be viewed as a proof of concept that an Internet version of the TCI can be used as a measure of personality in studies investigating the effect of psychological treatments delivered via the Internet. As pointed out by Andersson (2010), there is a current tendency that novel CBT protocols are tested in Internet trials soon after that they have been developed and that new psychological treatments are developed directly for use with the Internet (e.g. Ljótsson et al., 2010). Including TCI in future Internet trials may provide an opportunity to conduct large-scale studies, investigating possible working mechanisms of psychological treatments. In addition, this study format opens up for large studies that could investigate how TCI dimensions could predict outcome in psychological treatments (Spek et al., 2008).

## Conclusions

This study showed that an Internet-delivered cognitive behavioral treatment had an effect on Harm Avoidance and Self-Directedness, on par with other treatments for depression. There were larger changes in Harm Avoidance and Self-Directedness among responders compared to non-responders. The effect seems to be explained by changes in depressive states, rather than by the ICBT treatment itself.
